# The impact of article processing charge waiver on conducting research in low-income countries

**DOI:** 10.1186/s13031-021-00413-1

**Published:** 2021-10-15

**Authors:** M-Nasan Abdul Baki, Mahmoud Alhaj Hussein

**Affiliations:** University of Hama College of Human Medicine, Hama, Syria

**Keywords:** Syria, Article processing charge, Waiver, Low-income countries, Research

## Abstract

Health sciences research is a major tool in exchanging publications and knowledge between the various countries of the world. Researchers in developing countries barely have any financial funding from governmental or educational institutes to support their research. In low-income countries such as Syria, With less than 30$ per month and almost no financial support, Syrian residency doctors are fighting to push the scientific research reality of this ongoing crisis country forward and without a doubt, APC waiver plays a crucial role in this continuing mission.

Health Scientific research plays a crucial role in the never-stopping advancement of public health and clinical care. In order to achieve the maximum benefit from scientific research, the science gap between developed and developing countries should be closed. The cost of publishing research is one of the most important problems facing researchers from developing countries,especially article-processing charges (APCs) which can vary from hundreds to thousands of USD [1].

Since 2011, the ongoing crisis in Syria has had a massive effect on the lives of Syrians and made substantial damage to infrastructure, leading to a severe shortage of health facilities, a deteriorating economic situation, as well as the immigration of minds disrupting almost all aspects of life. Despite all the obstacles faced by Syrian medical researchers, there has been an increase in the amount of Syrian medical, dental, and pharmaceutical publications in the last decade [2].

To cover article-processing charges (APCs), authors tend to make use of funding that grants from governmental and educational institutes.Otherwise, researchers from Syria barely have any support and they have to conduct their research with limited funding and poor infrastructure [3]. Moreover, a salary of 73,000 Syrian pounds (SYP) per month equivalent to less than 30 USD, might be the only income for doctors throughout their residency years [4], thus it may take years to bear the costs of publishing a single scientific paper.

As a result, Syrian researchers tend to rely on the APC waivers granted by many journals to papers whose authors are based in countries classified by the World Bank as low-income economies [5].

Finally, APC waiver has a great impact on preserving the ongoing publications conducted in low-income countries such as Syria. We emphasize that the world should take real steps toward supporting authors from developing countries, which could be achieved by increasing the number of journals offering the waiver and encouraging the international and local health organizations to provide financial support. Hence, enhancing the endeavor to melt the scientific barriers between the different countries of the world.

## Abbreviations

APC: Article processing charge
USD: United States dollar
